# Genotypic and phenotypic diversity of polyhydroxybutyrate (PHB) producing *Pseudomonas putida* isolates of Chhattisgarh region and assessment of its phosphate solubilizing ability

**DOI:** 10.1007/s13205-014-0198-9

**Published:** 2014-02-19

**Authors:** Toshy Agrawal, Anil S. Kotasthane, Renu Kushwah

**Affiliations:** 1 Department of Plant Molecular Biology & Biotechnology, Indira Gandhi Krishi Vishwavidyalaya, Krishak Nagar, Raipur, 492006 Chattisgarh India; 2 Department of Plant Pathology, Indira Gandhi Krishi Vishwavidyalaya, Krishak Nagar, Raipur, 492006 Chattisgarh India

**Keywords:** Diversity, Phosphate solubilization, Polyhydroxybutyrate, Pseudomonas

## Abstract

A diverse and versatile spectrum of metabolic activities among isolates of fluorescent *Pseudomonas putida* indicates their adaptability to various niches. These polyhydroxybutyrate producing and phosphate solubilizing isolates showed a high level of functional and genetic versatility among themselves. One of the potential *P. putida* isolate P132 can contribute as a candidate agent for both biocontrol and PGPR applications. Identified as one of the most efficient PHB producer and phosphate solubilizer, in vitro detection of P132 showed the presence of genes for phenazine, pyrrolnitrin, pyoluteorin and 2,4 diacetylphloroglucinol along with polyhydroxyalkanoate.

## Introduction

*Pseudomonas putida* are ubiquitous bacteria frequently present in water, in soils, and especially in the plant rhizosphere (Timmis 2002; Dos Santos et al. 2004). These aerobic, gram-negative Pseudomonads possess many traits that make them well suited as biocontrol and growth promoting agents (Weller 1988; Lemanceau 1992; Weller et al. 2002; Fravel 2005). A large number of secondary metabolites (Leisinger and Margraff 1979), growth hormones (Brown 1972), antibiotics (Fravel 1988, 2005; Weller et al. 2002) and chelating compounds such as siderophores (Leong 1986) are known to be released by these fluorescent Pseudomonads. Some of them may also be involved in the biodegradation of natural or man-made toxic chemical compounds (Holloway 1992; Ramos et al. 2009). *P. putida* show diverse spectrum of metabolic versatility and niche-specific adaptations (Rojo 2010; Wu et al. 2011).

There may be direct or indirect mechanisms of these rhizobacteria as plant growth promoters and biological control agents. Phosphate solubilization is one of the direct mechanisms (Rodríguez and Fraga 1999; Mayak et al. 2004; Shahzad et al. 2010) and production of antibiotics such as 2,4-diacetyl phloroglucinol (DAPG), phenazine, pyoluteorin and pyrrolnitrin against pathogenic fungi and bacteria is among indirect mechanisms of PGPR (Ramamoorthy et al. 2001; McSpadden Gardener B 2008). Apart from primary and secondary metabolite production, certain fluorescent Pseudomonads (especially *P. putida*) are suitable as whole-cell biocatalyzers for the production of several value-added industrial compounds such as biodegradable and biocompatible polyesters called polyhydroxyalkanoates (PHA) or polyhydroxybutyrates (PHB). It accumulates as discrete granules and is used as storage material for carbon and for reducing equivalents by *P. putida.* This property has been widely exploited for their targeted biosynthesis in this organism (Hoffmann and Rehm 2004). Different strains of *P. putida* such as *P. putida* KT2440, *P. putida* GPo1, *P. putida* S12, etc. have been investigated for its capacity to accumulate PHAs and PHBs from different carbon sources (Durner et al. 2001; Hartmann et al. 2004; Kim et al. 2007; Meijnen et al. 2008). The pha gene cluster is responsible for the accumulation of PHAs and PHBs in *P. putida* (Chung et al. 2009; Vo et al. 2008; Wang and Nomura 2010).

PCR ribotyping has been used to characterize *Pseudomonas* spp. which concentrate on the analysis of the segments of the ribosomal genes and discriminate between isolates of same species by use of their chromosomal differences. The three main sets of repetitive elements used for typing purposes are the repetitive extragenic palindromic (REP) sequence, the enterobacterial repetitive intergenic consensus sequence (ERIC) and the BOX elements. Additionally, the availability of antibiotic gene sequences has enabled design of primers based on conserved regions for polymerase chain reaction (PCR) detection of antibiotic-producing bacteria (Raffel et al. 1996; Raaijmakers et al. 1997; Bangera and Thomashow 1999; McSpadden Gardener et al. 2001; de Souza and Raaijmakers 2003).

Multidisciplinary application of fluorescent Pseudomonads makes it significant and essential to study their phenotypic diversity along with genotypic variability. This will be helpful in designing strategies to use the indigenous isolates as bio-inoculants for biocontrol as well as plant growth promotion. This offers a viable substitute for the use of chemical inputs in agriculture. However, an effective biological control strain isolated from one region may not perform effectively in other soils or plants. Therefore, in an attempt to study the diversity of indigenous fluorescent Pseudomonads in Chhattisgarh, a large number of fluorescent Pseudomonads were isolated from the forest and agricultural soils, characterized and maintained in the Department of Plant Molecular Biology and Biotechnology, Indira Gandhi Krishi Vishwavidyalaya, Raipur. The purpose of present investigation was to assess the representative *P. putida* isolates for their polyhydroxybutyrate production and phosphate solubilizing ability using an array of in vitro assays; metabolite utilization tests and genotypic profiling were performed with species-specific ERIC primers and some antibiotic gene-specific primers. Species-specific primer was used for taxonomic affiliation of *P. putida.* The overall aim of the present investigation was thus to exploit and have a better understanding of beneficial activities of *P. putida* isolates.

## Materials and methods

### Bacterial isolates

The experimental material consisted of purified twenty-four isolates of *P. putida* isolated from soil samples of different geographical locations of Chhattisgarh as listed in Table 1. Both rhizospheric and non-rhizospheric soil samples were collected and used for isolation of fluorescent pseudomonads by adopting serial dilution method in King’s B medium. After incubation at 28 °C for 2 days, fluorescent pseudomonad colonies from plates were identified under UV light (366 nm). Isolates were characterized on the basis of biochemical tests as per the procedures outlined in Bergey’s Manual of Systematic Bacteriology (Sneath et al. 1986). Purified single colonies were further streaked onto KB agar plates to obtain pure cultures. The isolates were maintained in the culture collections of the Department of Plant Molecular Biology and Biotechnology, Indira Gandhi Krishi Vishwavidyalaya, Raipur, Chhattisgarh, India. Bacterial cultures were maintained at −80 °C on King’s B broth (Himedia) containing 50 % (w/v) glycerol and revived on King’s B slants as per requirement.

**Table 1:** *Pseudomonas putida* isolates used in the present study

S. no.	Isolates	Origin/location
1	P2	Cowpea soil, IGKV Horticulture garden, Raipur
2	P3	Fallow land soil, IGKV Horticulture garden, Raipur
3	P7	Fenugreek soil, IGKV Horticulture, Raipur
4	P23	Termitorium soil, VIP Road, Raipur
5	P29	Termitorium soil, VIP Road, Raipur
6	P43	Abhanpur road
7	P45	Abhanpur road
8	P56	Bhatagaon
9	P59	Bhatagaon (degraded paddy straw)
10	P74	Chhati
11	P80	Darba
12	P123	Kanker forest-2
13	P130	Kanker forest-3
14	P132	Kanker forest-3
15	P144	Rice field, Kodebor
16	P150	Kurud
17	P163	Nursery, Raipur
18	P166	Purur
19	P174	Rice field, Rajiv Gandhi Marg, Raipur
20	P184	Arhar-rice field soil, Satpara
21	P187	Arhar-rice field soil, Satpara
22	P191	Forest soil, Raipur
23	P192	Forest soil, Raipur
24	P207	Bamboo soil, VIP road, Raipur

### Phenotypic characterization of *P. putida* isolates

The phenotypic characterization of *P. putida* isolates were done on the basis of fluorescence on King’s B (KB) medium, gelatin liquefaction, casein hydrolysis, lipolytic activity, nitrate reduction, growth at 4 and 42 °C, oxidase test, phenylalanine test and egg yolk medium test (Stanier et al. 1966; Holt et al. 1994). A rapid antibiotic sensitivity test was used to distinguish different species of fluorescent *Pseudomonas*. Antibiotic sensitivity studies were performed by the streak plate method of Bauer et al. (1966). Kanamycin and carbenicillin sensitivity was determined by incorporating 1 mg/ml of Kanamycin and 0.1 mg/ml of Carbenicillin, respectively, in King’s B medium. *Pseudomonas* spp. showing positive growth on either of the antibiotic supplemented medium was resistant.

Hicarbohydrate™ kit was used to test carbon utilization profiles as described by the manufacturer (Himedia Laboratories, Mumbai, India). Cells were grown in King’s medium B broth to reach density of 0.5 O.D. at 600 nm. An aliquot of 50 μl of this suspension was inoculated to each well of Hicarbohydrate™ kit, incubated at 30 °C for 24 h and the results were registered according to the instructions of the manufacturer. The experiment was done with three replicates.

### Screening for polyhydroxybutyrate (PHB) production and its quantitative estimation

*Pseudomonas putida* isolates were screened for PHB accumulation qualitatively by following the viable colony method using Sudan Black B dye (Juan et al. 1998). Sterilized Nutrient agar (Himedia) supplemented with 1 % glucose was spot inoculated with the isolates and incubated at 30 °C for 24 h. Ethanolic solution (0.02 %) of Sudan Black B was spread over the colony and the plates were kept undisturbed for 30 min. Later, they were washed with ethanol (96 %) to remove the excess stain from the colony. The dark blue colored colony was taken as positive for PHB production.

The Sudan Black B positive isolates were subjected to quantification of PHB production as per the method of John and Ralph (1961). The bacterial cells containing the polymer were pelleted at 10,000 rpm for 10 min. and the pellet was washed with acetone and ethanol to remove the unwanted materials. The pellet was resuspended in equal volume of 4 % sodium hypochlorite and incubated at room temperature for 30 min. The whole mixture was again centrifuged and the supernatant discarded. The cell pellet containing PHB was again washed with acetone and ethanol. Finally, the polymer granules were dissolved in hot chloroform. The chloroform was filtered and to the filtrate, concentrated 10 ml hot H_2_SO_4_ was added. The addition of sulfuric acid converts the polymer into crotonic acid which is brown colored. The solution was cooled and the absorbance read at 235 nm against a sulfuric acid blank. By referring to the standard curve prepared using Poly[(*R*)-3-hydroxybutyric acid] (Sigma Aldrich, USA) by following the method of Law and Slepecky (1969), the quantity of PHB produced by different bacterial isolates was determined.

### Screening of phosphate solubilisation ability and its quantitative estimation

Qualitative screening of phosphate solubilising *P. putida* was performed on Pikovskaya agar medium (Himedia) containing tricalcium phosphate as a phosphate source and bromocresol purple (0.1 g/l) as a pH indicator for acidification (Vazquez et al. 2000). After incubation of fresh cultures of *P. putida* at 28 ± 2 °C for 48 h, phosphate solubilising isolates turned the media color from purple to yellow in the zones of acidification.

Quantitative estimation of phosphate solubilisation in Pikovskaya broth (Himedia) was performed according to the procedure of Murphy and Riley (1962). Fresh cultures of *P. putida* isolates were inoculated to 50 ml of Pikovskaya’s broth and incubated at 28 ± 2 °C and 100 rpm. The amount of inorganic phosphate (Pi) released in the broth was estimated after 7 days of incubation in comparison with un-inoculated control. The broth culture was centrifuged at 10,000 rpm for 10 min to separate the supernatant from the bacterial growth and insoluble phosphate. To the 0.5 ml of the culture supernatant 5 ml of chloromolybdic acid was added and mixed thoroughly. Volume was made up to 10 ml with distilled water and 125 μl of chlorostannous acid was added to it. Immediately, the final volume was made up to 25 ml with distilled water and mixed thoroughly. After 15 min, the blue color developed was read in a spectrophotometer at 610 nm using a reagent blank. Corresponding amount of soluble phosphorous was calculated from standard curve of potassium dihydrogen phosphate (KH_2_PO_4_). Phosphate solubilizing activity was expressed in terms of tricalcium phosphate solubilization which in turn was measured by μg/ml of available orthophosphate as calibrated from the standard curve of KH_2_PO_4_.

### 16S rRNA gene amplification

Total genomic DNA from 24 *P*. *putida* isolates was extracted by the CTAB procedure (Ausubel et al. 1991) and used for amplification using various primers (Table 2). PCR primers designed from genes HI660468 (Pa49), HM067869 (Pa16S), HQ317190 (Pp16S), EF159157 (Pf16S) and AF869903 (Pf23S) were used to specifically distinguish species of *Pseudomonas* isolates by amplification of the nuclear rRNA gene cluster. These forward and reverse primers were designed using Batch primer3 software from following gene sequences: HI660468: Sequence 49 from Patent WO2010127969 of *Pseudomonas aeruginosa*, HM067869 of *Pseudomonas aeruginosa* strain GIM 32 16S ribosomal RNA gene, partial sequence, HQ317190 of *P. putida* strain DYJL49 16S ribosomal RNA gene, partial sequence, EF159157 of *Pseudomonas fluorescens* strain TNAUA2 16S ribosomal RNA gene and 16S–23S ribosomal RNA intergenic spacer, partial sequence and AF369903 of *Pseudomonas fluorescens* 23S ribosomal RNA gene, partial sequence procured from NCBI database. PCR was carried in 20 μl reaction mixture containing 1× assay buffer (10 mM Tris–HCl at pH 9.0, 50 mM KCl, 2.5 mM MgCl_2_), 0.1 mM each dNTP mix, 1 μM both forward and reverse primers, 60–90 ng of template DNA and 0.5 U Taq DNA polymerase (Axygen) in a programmable thermo cycler (M/s Biorad Laboratories India Pvt. Ltd) according to the following thermo-cycling conditions: 95 °C for 5 min, 35 cycles of 1 min at 95 °C, 60 °C for 1 min, 72 °C for 1 min and final elongation step at 72 °C for 7 min.

**Table 2:** Details of PCR primers used in the present study

S. no.	Primers	Gene/reference	Sequence(5′–3′)	Length	Expected product size (bp)
1	Pa49-F	HI660468	TCTTCCGCCTGTTCAATTACCCGA	24	448
	Pa49-R		AATACCTTGGCCACCTTGTTCAGC	24	
2	Pa16S-F	HM067869	AGAGGGTGGTGGAATTTCCTGTGT	24	586
	Pa16s-R		TACCGACCATTGTAGCACGTGTGT	24	
3	Pp16S-F	HQ317190	ACCGACAGAATAAGCACCGGCTAA	24	364
	Pp16S-R		AAGAGTTCAAGACTCCCAACGGCT	24	
4	Pf16S-F	EF159157	TCCCTATCGATTGATCCGGCTTCT	24	250–260
	Pf16S-R		TTTAGATGGTGGAGCCAAGGAGGA	24	
5	Pf23S-F	AF869903	ACGCTTTCTTTAAAGGGTGGCTGC	24	400–420
	Pf23S-R		TCTATCCATGGGCAGGTTGAAGGT	24	
6	ERIC-F	de Bruijn (1992)	AAGTAAGTGACTGGGGTGAGCG	22	
	ERIC-R		TATAAGCTCCTGGGGATTCAC	21	
7	PhaJ1-F	Polyhydroxyalkanoate	AAGGCCGAGTACAAGAAGTCCGTT	24	240–250
	PhaJ1-R		TCACCGGTTTCTGGAAGCTCATCT	24	
8	PHZ1	Phenazine	GGCGACATGGTCAACGG	17	1,400
	PHZ2	Delaney et al. (2001)	CGGCTGGCGGCGTATAT	17	
9	PCA2a	Phenazine	TTGCCAAGCCTCGCTCCAAC	20	1,400
	PCA3B	Raaijmakers et al. (1997)	CCGCGTTGTTCCTCGTTCAT	20	
10	B2BF	2,4 Diacetyl phloroglucinol	ACCCACCGCAGCATCGTTTATGAGC	25	~470 or 629
	BPR4	McSpadden Gardener et al. (2001)	CCGCCGGTATGGAAGATGAAAAAGTC	26	
11	PrnAF	Pyrrolnitrin	GTGTTCTTCGACTTCCTCGG	20	1,050
	PrnAR	de Souza and Raaijmakers (2003)	TGCCGGTTCGCGAGCCAGA	19	
12	phlA-1f	2,4 Diacetyl phloroglucinol	TCAGATCGAAGCCCTGTACC	20	418
	phlA-1r	Rezzonico et al. (2003)	GATGCTGTTCTTGTCCGAGC	20	
13	plt1	Pyoluteorin	ACTAAACACCCAGTCGAAGG	20	~440
	plt2	Mavrodi et al. (2001)	AGGTAATCCATGCCCAGC	18	
14	PrnCf	Pyrrolnitrin	CCACAAGCCCGGCCAGGAGC	20	~720
	PrnCr	Mavrodi et al. (2001)	GAGAAGAGCGGGTCGATGAAGCC	23	
15	Phl2a	2,4 Diacetyl phloroglucinol	GAGGACGTCGAAGACCACCA	20	~745
	Phl2b	Raaijmakers et al. (1997)	ACCGCAGCATCGTGTATGAG	20	
16	PltBf	Pyoluteorin	CGGAGCATGGACCCCCAGC	19	~700 or 900
	PltBr	Mavrodi et al. (2001)	GTGCCCGATATTGGTCTTGACCGAG	25	

### ERIC-PCR-based genotypic analysis

ERIC primer sequences were used in PCR to detect differences in the number and distribution of these bacterial repetitive sequences in the bacterial genome. ERIC-PCR was carried out using the primer sequences ERIC-F (5′AAGTAAGTGACTGGGGTGAGCG3′) and ERIC-R (5′TATAAGCTCCTGGGGATTCAC3′) as described by de Bruijn (1992). ERIC-PCR was carried in 20 μl reaction mixture containing 1× assay buffer (10 mM Tris–HCl at pH 9.0, 50 mM KCl, 2.5 mM MgCl_2_), 0.1 mM each dNTP mix, 1 μM both forward and reverse primers, 60–90 ng of template DNA and 1 U Taq DNA polymerase (Axygen) in a programmable thermo cycler (M/s Biorad Laboratories India Pvt. Ltd) according to the following thermo-cycling conditions: 94 °C for 3 min, 45 cycles of 45 s at 94 °C, 53 °C for 1 min, 72 °C for 1 min and final elongation step at 72 °C for 8 min.

### In vitro detection of antibiotic-producing *P. putida* isolates using gene-specific primers

Primers (Imperial Life Sciences) for the different PCR-based screening of genes that encode for antibiotics are detailed in Table 2. Preparation of bacterial templates for detecting antibiotic-producing genes was carried out as described by Wang et al. (2001) and Rezzonico et al. (2003). PCR amplification of primers PhaJ1F-R, PHZ1-2, PCA2a-3B, B2BF-BPR4, PrnAF-R, phlA-1f-r, plt1-2, PrnCf-r, Phl2a-2b and PltBf-r was carried out in 20 μl reaction mixtures containing 3 μl of lysed bacterial suspension, 1X assay buffer (10 mM Tris–HCl at pH 9.0, 50 mM KCl, 2.5 mM MgCl_2_), 0.4 mM dNTPs, 1 μM of each primer and 1 U of Taq DNA polymerase (Axygen). Amplification was performed in a programmable thermo cycler (M/s Biorad Laboratories India Pvt. Ltd). The cycling program for PCA2a-3B, PrnAF-R, plt1-2, PrnCf-r, PHZ1-2, Phl2a-2b, PhaJ1F-R, and PltBf-r included an initial denaturation at 95 °C for 3 min followed by 35 cycles of 95 °C for 1 min, 62 °C (for PCA2a-3B, PrnAF-R, plt1-2, PrnCf-r, PHZ1-2)/52 °C (for Plt1-2)/60 °C (for Phl2a-2b, PhaJ1F-R)/65 °C (for B2BF-BPR4) for 1 min, 72 °C for 1 min, and then a final extension at 72 °C for 5 min. However, the cycling program for phlA-1f-r included an initial denaturation at 94 °C for 5 min followed by 35 cycles of 94 °C for 30 s, 62 °C for 30 s, 72 °C for 45 s, and then a final extension at 72 °C for 5 min.

The amplification products were electrophoresed in a 1 % (w/v) agarose gel with 1× TBE buffer at 80 V at room temperature, stained with ethidium bromide and photographed under UV light by Biorad Gel-Documentation as well as on 5 % native polyacrylamide gel (visualized by silver staining).

### Statistical analysis

All the experiments were conducted in three completely randomized replicates. On the basis of data derived from the carbon source utilization profiles, a matrix with binary code composing positive (1) and negative (0) values was made. SIMQUAL program was used to compute the symmetric matrix in the form of average taxonomic distances. Sequential, agglomerative, hierarchical and nested (SAHN) clustering was used for the cluster analyses. Phenogram was constructed from the similarity matrix by the un-weighted pair group with mathematical averages (UPGMA) using NTSYS-pc2.02a (Exeter software, New York, USA) numerical taxonomy and multivariate analysis system. Similar method was followed to construct dendrogram using binary data of ERIC primer-based PCR amplification of *P. putida* isolates.

Replicated data of quantitative estimation of PHB production and P solubilization of all the 24 *P. putida* isolates were subjected to statistical analysis using WASP (Web Agri Stat Package) software (http://icargoa.res.in/wasp/index.php). Critical difference at 0.05 level of significance was calculated for the observed values along with average and standard deviation. Duncan’s test controls the Type I comparison wise error rate and as per Duncan’s grouping mean values with the same letter are not significantly different.

## Results and discussion

### Phenotypic characterization of *P. putida* isolates

Isolates were characterized on the basis of biochemical and antibiotic sensitivity tests. All the isolates of *P. putida* were positive for cytochrome oxidase. Isolates showed variability for traits such as gelatin liquefaction, casein hydrolysis, lipolytic activity, nitrate reduction and antibiotic sensitivity test. Of the 24 *P. putida*, 9 isolates (37.5 %) showed proteolytic activity (casein hydrolysis) by inducing clear zones around the cells on skim milk agar medium, 7 isolates (29.17 %) showed lipolytic activity, 14 isolates (58.33 %) were negative for nitrate test and 10 isolates (41.67) gave positive result for nitrate test (of which 6 isolates P23, P43, P59, P80, P144 and P174 were positive before addition of zinc and 4 isolates P187, P191, P192 and P207 showed positive response after addition of zinc). Only three isolates P56, P130 and P191 were positive for phenylalanine test as indicated by appearance of green color after addition of few drops of 10 % aq. ferric chloride to the cultures grown in phenyl alanine amended medium. Lecithinase production was observed as opaque precipitate around colonies of four *P. putida* isolates viz. P59, P80, P123 and P166 resulting in lecithin positive result in egg yolk medium. Blazevic et al. (1973) reported some diagnostic tests for differentiation of *P. fluorescens* and *P. putida* isolates. *P. aeruginosa* is the only fluorescent pseudomonads that can grow at 42 °C whereas *P. fluorescens* grow at 4 °C. In the present investigation none of the isolates showed growth at 4 °C and 42 °C. A shortened gelatin test can differentiate *P. fluorescens* (positive) from *P. putida* (negative). Present result correlated with this fact except the isolates P2, P3, P45, P144, P192 and P207 which liquefies gelatin. *P. fluorescens* and *P. putida* are very sensitive to low levels of kanamycin and resistant to carbenicillin, a pattern just the opposite of that obtained with *P. aeruginosa*. All the isolates were resistant to antibiotic carbenicillin and sensitive to kanamycin. However, isolates P56 and P174 were tolerant to both the antibiotics (Table 3). Several strains within the family *Pseudomonadaceae* such as *P*. *putida* S12 show significant intrinsic resistance to multiple antibiotics (Kieboom and de Bont 2001). Blazevic et al. (1973) further suggested that the shortened test for nitrate reduction, then, together with the marked sensitivity to kanamycin and resistance to carbenicillin would provide a rapid means of accurately identifying *P. fluorescens* and *P. putida* and separating them from *P. aeruginosa*. However, rare nitrate-negative *P. aeruginosa* or rare nitrate-positive *P. fluorescens* or *P. putida* should not be misidentified using both of these characteristics.

**Table 3:** Differential phenotypic characteristics revealed by twenty-four *P. putida* isolates

Tests	P2	P3	P7	P23	P29	P43	P45	P56	P59	P74	P80	P123
1	−	−	−	−	−	−	−	−	−	−	−	−
2	+	+	+	+	+	+	+	−	+	+	+	+
3	−	−	−	−	−	−	−	−	−	−	−	−
4	−	−	−	−	−	−	−	−	−	−	+	−
5	+	+	+	+	+	+	+	+	+	+	+	+
6	+	+	+	+	+	+	+	−	+	+	+	+
7	−	−	−	−	−	−	−	−	−	−	−	−
8	−	−	−	+	−	−	−	−	−	−	+	−
9	+	+	+	+	+	+	+	−	+	+	+	+
10	−	−	−	−	−	−	−	−	±	−	±	−
11	±	−	−	+	−	−	−	−	+	+	+	+
12	+	+	+	+	+	+	+	−	+	+	+	+
13	−	−	−	−	−	−	−	−	−	−	−	−
14	−	−	−	−	−	−	−	−	−	−	−	−
15	−	−	−	−	−	−	−	−	−	−	−	−
16	−	−	±	−	−	−	−	−	−	−	−	−
17	−	−	−	−	−	−	−	−	−	−	−	−
18	−	−	−	−	−	−	−	−	−	−	−	−
19	−	−	−	−	−	−	−	−	−	−	−	−
20	−	−	−	−	−	−	−	−	−	−	−	−
21	−	−	−	−	−	−	−	−	−	−	−	−
22	−	−	−	−	−	−	−	−	−	−	−	−
23	−	−	−	−	−	−	−	−	−	−	−	−
24	−	−	−	−	−	−	−	−	−	−	−	−
25	−	−	−	−	−	−	−	−	−	−	−	−
26	−	−	−	−	−	−	−	−	−	−	−	−
27	−	−	−	−	−	−	−	−	−	−	−	−
28	−	−	−	−	−	−	−	−	−	−	−	−
29	+	+	±	+	+	+	+	−	+	+	+	+
30	−	−	−	−	−	−	−	−	−	−	−	−
31	+	+		+	+	+	+	+	+	+	+	+
32	−	−	−	−	−	−	−	−	−	−	−	−
33	+	+	+	+	+	+	+	+	+	+	+	+
34	+	+	+	−	+	+	+	+	−	−	+	+
35	−	−	−	−	−	−	−	−	−	−	−	−
36	−	+	−	+	+	−	−	−	−	−	−	−
37	+	+	−	−	−	−	+	−	−	−	−	−
38	−	−	−	+	−	+	−	−	−	−	−	−
39	+	+	+	+	+	+	+	+	+	+	+	+
40	**−**	**−**	**−**	**−**	**−**	**−**	**−**	+	**−**	**−**	**−**	**−**
41	**−**	**−**	**−**	**−**	**−**	**−**	**−**	**−**	L+	**−**	L+	L+
42	**−**	**−**	**−**	**−**	**−**	**−**	**−**	**−**	**−**	**−**	**−**	**−**
43	**−**	**−**	**−**	**+**	**−**	**+**	**−**	**−**	**+**	**−**	**+**	**−**
44	**−**	**−**	**−**	NA	**−**	NA	**−**	**−**		**−**	NA	**−**
45	R	R	R	R	R	R	R	R	R	R	R	R
46	S	S	S	S	S	S	S	R	S	S	S	S

Tests	P130	P132	P144	P150	P163	P166	P174	P184	P187	P191	P192	P207
1	−	−	−	−	−	−	−	−	−	−	−	−
2	+	+	+	+	+	+	+	+	+	+	+	+
3	−	−	−	−	−	−	−	−	−	−	−	−
4	−	−	−	−	−	−	−	−	−	+	−	−
5	+	+	+	+	+	+	+	+	+	+	+	+
6	+	+	+	+	+	+	+	+	+	+	+	+
7	−	−	−	−	−	−	−	−	−	−	−	−
8	−	−	−	−	−	−	−	−	−	−	−	−
9	+	+	+	+	+	+	+	+	+	+	+	+
10	−	−	−	−	−	−	−	−	−	−	−	−
11	+	+	+	+	+	+	+	+	+	+	+	+
12	+	+	+	+	+	+	+	+	+	+	+	+
13	−	−	−	−	−	−	−	−	−	−	−	−
14	−	−	−	−	−	−	−	−	−	−	−	−
15	−	−	−	−	−	−	−	−	−	−	−	−
16	−	−	−	−	−	−	−	−	−	−	−	−
17	−	−	−	−	−	−	−	−	−	−	−	−
18	−	−	−	−	−	−	−	−	−	−	−	−
19	−	−	−	−	−	−	−	−	−	−	−	−
20	−	−	−	−	−	−	−	−	−	±	−	−
21	−	−	−	−	−	−	−	−	−	±	−	−
22	−	−	−	−	−	−	−	−	−	+	−	−
23	−	−	−	−	−	−	−	−	−	−	−	−
24	−	−	−	−	−	−	−	−	−	−	−	−
25	−	−	−	−	−	−	−	−	−	−	−	−
26	−	−	−	−	−	−	−	−	−	−	−	−
27	−	−	−	−	−	−	−	−	−	−	−	−
28	−	−	−	−	−	−	−	−	−	−	−	−
29	+	+	+	±	+	+	+	+	+	+	+	+
30	−	−	−	−	−	−	−	−	−	−	−	−
31	+	+	+	+	+	+	+	+	+	+	+	+
32	−	−	−	−	−	−	−	−	−	−	−	−
33	+	+	+	+	+	+	+	+	+	+	+	+
34	+	+	+	+	+	+	+	−	+	+	+	+
35	−	−	−	−	−	−	−	−	−	−	−	−
36	−	−	+	−	−	+	−	−	+	+	+	+
37	−	−	+	−	−	−	−	−	+	−	+	+
38	−	−	+	−	−	−	−	−	+	+	+	+
39	+	+	+	+	+	+	+	+	+	+	+	+
40	+	−	−	−	−	−	−	−	−	+	−	−
41	−	−	−	−	−	L+	−	−	−	−	−	−
42	−	−	−	−	−	−	−	−	−	−	−	−
43	−	−	+	−	−	−	+	−	NA	NA	NA	NA
44	−	−		−	−	−	NA	−	+	+	+	+
45	R	R	R	R	R	R	R	R	R	R	R	R
46	S	S	S	S	S	S	R	S	S	S	S	S

Different tests: 1, lactose; 2, xylose; 3, maltose; 4, fructose; 5, dextrose; 6, galactose; 7, raffinose; 8, trehalose; 9, melibiose; 10, sucrose; 11, l-arabinose; 12, mannose; 13, inulin; 14, sodium gluconate; 15, glycerol; 16, salicin; 17, dulcitol; 18, inositol; 19 sorbitol; 20, mannitol; 21, adonitol; 22, arabitol; 23, erythritol; 24, α-methyl-d-mannoside; 25, rhamnose; 26, cellobiose; 27, melezitose; 28, α-methyl-d-mannoside; 29, xylitol; 30, ONPG; 31, esculin hydrolysis; 32, d-arabinose; 33, citrate utilization; 34, malonate utilization; 35, sorbose; 36, casein hydrolysis; 37, gelatin hydrolysis; 38, lipase test; 39, oxidase test; 40, phenyl alanine test; 41, egg yolk reaction; 42, growth at 4 °C/42 °C; 43, nitrate test (before adding Zn); 44, nitrate test (after adding Zn); 45, carbenicillin sensitivity; 46, kanamycin sensitivity. +, Positive reaction; −, negative reaction; ±, partially positive; L+, lecithinase positive; R, resistant; S, susceptible; NA, not applicable

**Fig. 1:**
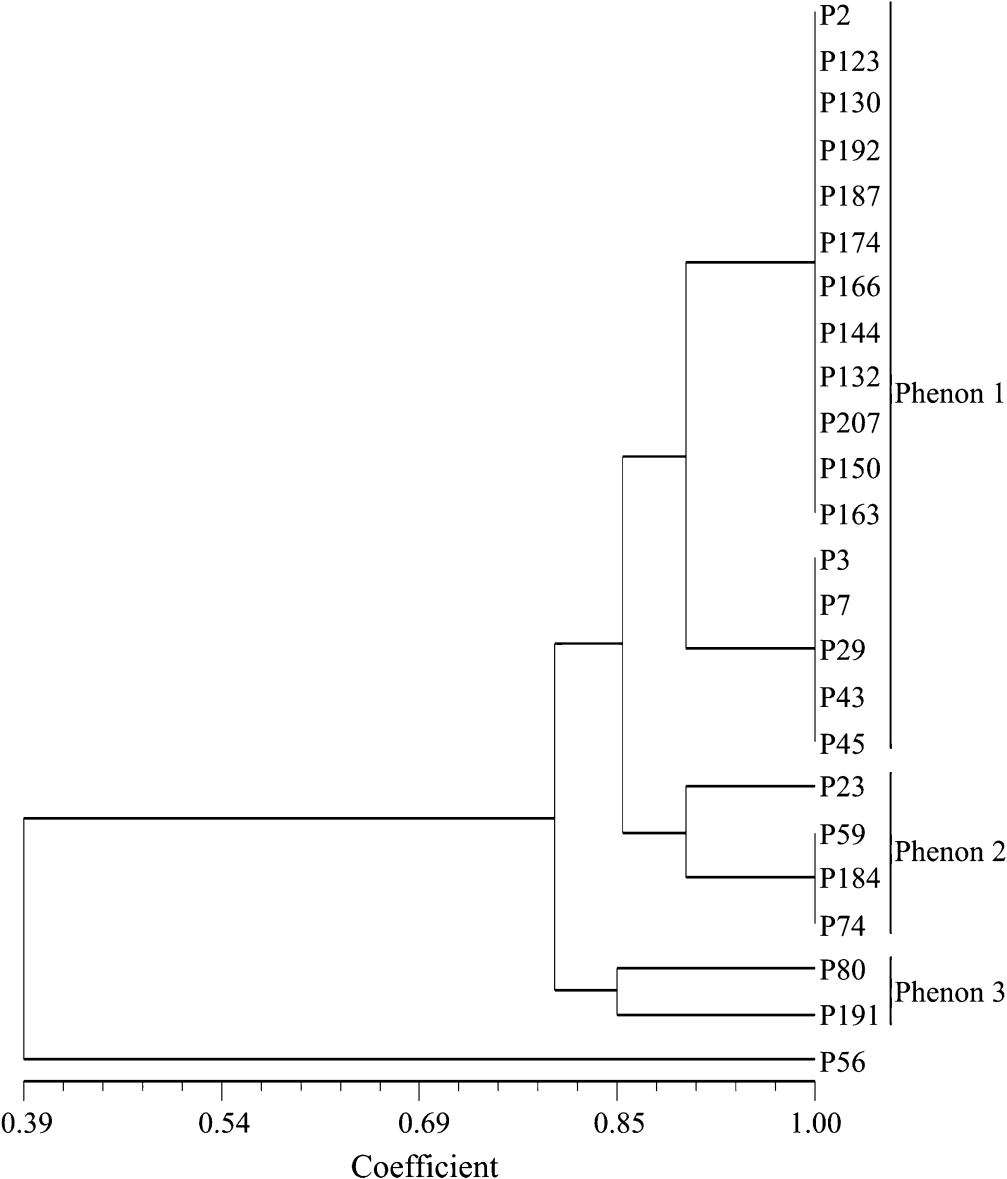
Phenogram of 24 *P. putida* isolates based on their carbon source utilization profiles

All the *P. putida* isolates utilized xylose, dextrose, galactose, melibiose, mannose, xylitol, esculin and citrate but exhibited varying degree of utilization towards other carbon sources such as fructose, trehalose, l-arabinose, arabitol and malonate. These isolates did not utilize lactose, maltose, raffinose, sucrose, inulin, sodium gluconate, glycerol, salicin, dulcitol, inositol, sorbitol, mannitol, adonitol, arabitol, erythritol, α-methyl-d-gluconate, rhamnose, cellobiose, melezitose, α-methyl-d-mannoside, ONPG, d-arabinose and sorbose. Isolate P56 did not utilize xylose, galactose, melibiose, mannose and xylitol which were utilized by all other 23 *P. putida* isolates (Table 3). Numerical analysis of phenotypic characteristics revealed polymorphism among *P. putida* isolates. All 24 *P. putida* isolates were grouped into 3 major phenons at 0.76 similarity coefficient level (Fig. 1). The similarity coefficient range among 24 *P. putida* isolates was 0.39–1.00. Phenons 1, 2 and 3 consist a total of 17, 4 and 2 isolates, respectively. Isolate P56 did not fall into any of the phenogram and revealed all together distinct identity. Differential utilization of carbon sources by isolates of *P. putida* as identified by Hi-carbohydrate™ kit test may play an important role in adapting to a variety of crop plants and soil types. Xylose being second to glucose in natural abundance is a promising candidate substrate for bacterial growth (Beall et al. 1991). Carbohydrates serve as primary substrate for the synthesis of many important metabolites and commercial products by microorganisms. Meur et al. (2012) reported that sequential feeding of relatively cheap carbohydrates such as xylose is a practical way to achieve more cost-effective medium-chain-length (mcl) PHA production. Reduction in cost can be achieved using two kinds of carbon sources, one for biomass production and the other for synthesis of PHA. Metabolite utilization diversity is also important because changes in their composition may affect the patterns and activities of rhizobacterial populations which are dependent upon rhizospheric nutrients for growth. Broad spectrum carbon source utilization among *P. putida* isolates in the present study may help in developing and designing stimulators for specific application.

### Screening of polyhydroxybutyrate (PHB) producers and its quantification

Biodegradable and biocompatible polyesters such as polyhydroxyalkanoates (PHA) have potential pharmaceutical values (Takahashi et al. 1994). In an alkaline environment *P. putida* have been reported to produce medium-chain-length (R)-3-hydroxyalkanoates (Wang et al. 2007). In the present investigation all the 24 *P. putida* isolates gave positive result for PHB accumulation in Sudan Black B qualitatively screening test in glucose-supplemented nutrient agar medium. Madison and Huisman (1999) have also reported that these biopolymers are accumulated as inclusions (PHA granules) in the bacterial cytoplasm in response to inorganic nutrient limitations, generally, when the microbes are cultured in the presence of an excess carbon source. However, there was significant difference in quantitative analysis of all the isolates which ranged from 6.17 to 14.35 mg/ml. Variation was significant at both 0.01 and 0.05 levels. The lowest observed value was for isolate P130 and the highest was for isolate P2. Isolates P43, P56 and P187 also produced significantly higher amounts of PHB as compared to other isolates (Table 4). Expenditures for large-scale production of PHA are almost evenly divided among carbon source, fermentation process and separation process (Sun et al. 2007; Elbahloul and Steinbüchel 2009). Therefore screening for carbohydrate utilization of *P. putida* isolate may help in identifying candidate isolate which dwells upon cheaper carbon sources. Our work reports that all the 24 putida isolates utilized relatively cheaper carbohydrates such as xylose, dextrose, galactose, melibiose and mannose. PHB could be synthesized from a cheaper raw material xylose in *Pseudomonas pseudoflava* and *P. cepacia* up to 22 % (w/w) and 50 % (w/w), respectively (Bertrand et al. 1990; Young et al. 1994). Meur et al. (2012) also tested the growth of an engineered *P. putida* KT2440 strain in xylose to reduce the substrate cost for PHA production.

**Table 4:** PHB production and inorganic phosphate solubilization of *P. putida* isolates

S. no.	Isolates	PHB production (mg/ml)**	Phosphate solubilized (μg/ml)**
1	P2	14.35^a^ ± 0.45	57.38^j^ ± 11.85
2	P3	10.09^d,e,f,g^ ± 0.20	358.95^g^ ± 25.38
3	P7	10.39^c,d,e,f,g^ ± 1.19	651.95^a,b^ ± 29.62
4	P23	9.89^e,f,g^ ± 0.92	687.11^a^ ± 19.95
5	P29	9.51^g,h^ ± 0.67	566.12^c,d^ ± 45.42
6	P43	12.48^b^ ± 0.70	607.11^b,c,d^ ± 5.81
7	P45	7.98^h,i^ ± 0.82	260.56^h^ ± 44.63
8	P56	12.27^b^ ± 0.69	659.62^a,b^ ± 54.61
9	P59	11.59^b,c,d^ ± 0.73	478.11^f^ ± 31.26
10	P74	11.42^b,c,d,e,f^ ± 0.79	686.12^a^ ± 21.38
11	P80	11.38^b,c,d,e,f^ ± 1.02	607.11^b,c,d^ ± 22.78
12	P123	6.96^i,j^ ± 0.69	564.11^c,d^ ± 49.65
13	P130	6.17^j^ ± 1.15	626.45^a,b^ ± 30.33
14	P132	11.22^b,c,d,e,f^ ± 0.64	660.23^a,b^ ± 46.99
15	P144	11.48^b,c,d,e^ ± 0.72	594.67^b,c,d^ ± 8.02
16	P150	10.20^d,e,f,g^ ± 0.78	384.05^g^ ± 14.21
17	P163	6.81^i,j^ ± 0.76	582.68^c,d^ ± 27.82
18	P166	8.78^g,h^ ± 0.95	554.18^d,e^ ± 35.60
19	P174	9.35^g,h^ ± 0.88	494.89^e,f^ ± 8.33
20	P184	9.73^f,g^ ± 0.70	489.19^e,f^ ± 14.40
21	P187	12.15^b^ ± 1.09	653.98^a,b^ ± 35.32
22	P191	10.02^d,e,f,g^ ± 0.53	685.66^a^ ± 23.55
23	P192	11.98^b,c^ ± 1.14	548.23^d,e^ ± 55.48
24	P207	8.94^g,h^ ± 0.74	165.12^i^ ± 46.84
	Control		30.50^j^ ± 6.36
	CV	8.039	6.417
	CD (0.05)	1.695	66.910

Values are average of 3 replications; values after ± represents standard deviation

*CV* coefficient of variance, *CD* critical difference

** Values are significant at 1 and 5 % levels; As per Duncan’s grouping means with the same letter are not significantly different

Since isolates of *P. putida* are versatile and robust in catabolizing a broad range of compounds and resist adverse environmental conditions their metabolic repertoires funneled resources can be channeled towards PHA and PHB synthesis (Meijnen et al. 2009; Ciesielski et al. 2010; Poblete-Castro et al. 2012). The twenty-four *P. putida* used in the present investigation vary to some extent in their phenotypic behavior creating a broad range of industrial application possibilities. Its fast growth, high biomass yield and low maintenance demands are among key features for successful industrial application. With the current lifestyle, need of an hour is the production of eco-friendly plastic materials such as polyhydroxyalkanoic acids (PHAs) by rational, efficient and sustainable use of natural resources. The outcomes of the present study can be exploited for selection of potential PHB producer *P. putida* isolate for commercial application.

### Screening of phosphate solubilizers and its quantification

Phosphorus frequently is the least accessible macronutrient in many ecosystems and its low availability is often limiting to plant growth (Raghothama 1999). In vitro phosphate solubilization efficacy of *P. putida* isolates as performed on Pikovskaya agar by acidification showed positive results for all the 24 isolates tested. All the 24 isolates were capable of differentially utilizing tricalcium phosphate in both agar plate and broth assays. Quantitative estimation of soluble phosphate concentrations in Pikovskaya’s broth was expressed as μg/ml and it varied significantly from 57.38 to 687.11 μg/ml. Variation was significant at both 0.01 and 0.05 levels. The lowest value was observed for isolate P2 and highest for isolate P23. Phosphate solubilization by isolates P23, P74 and P191 was significantly highest among all the other isolates. All the rhizospheric isolates of *P. putida* showed variable phosphate solubilizing potential with P7, P23, P56, P74, P132, P187 and P191 being the best P solubilizers among all other 24 isolates releasing more than 650 μg/ml inorganic phosphate (Table 4). These candidate isolates can be used as microbial inoculants to improve soil fertility by releasing bound phosphorus thereby increasing the crop yield potential. The production of organic acids and acid phosphatases plays a major role in the mineralization of organic phosphorous in soil. Stimulation of different crops by plant growth promoting *P. putida* isolate(s) with potential phosphate solubilization ability may help in exploiting large reserves of phosphorus present in most agricultural soils. Inoculation of plants by a target pseudomonas at a much higher concentration than that normally found in soil is necessary because the numbers of several phosphate solubilizing bacteria already present in soil are not high enough to compete with other bacteria commonly established in the rhizosphere. However, study of ecological roles of these characterized phosphate solubilizers in soil is necessary for sustainable agricultural practices and commercial applications. Several *Pseudomonas* species have been reported among the most efficient phosphate solubilizing bacteria and as important bio-inoculants due to their multiple biofertilizing activities of improving soil nutrient status, secretion of plant growth regulators and suppression of soil-borne pathogens (Rodríguez and Fraga 1999; Gulati et al. 2008; Vyas et al. 2009). Genes from potential phosphate solubilizer *P. putida* identified in the present investigation can be further exploited to study genetic regulation governing the mineral phosphate solubilization trait, which has an otherwise very less known information.

### 16S rRNA gene amplification and ERIC-PCR-based genotypic analysis

**Fig. 2:**
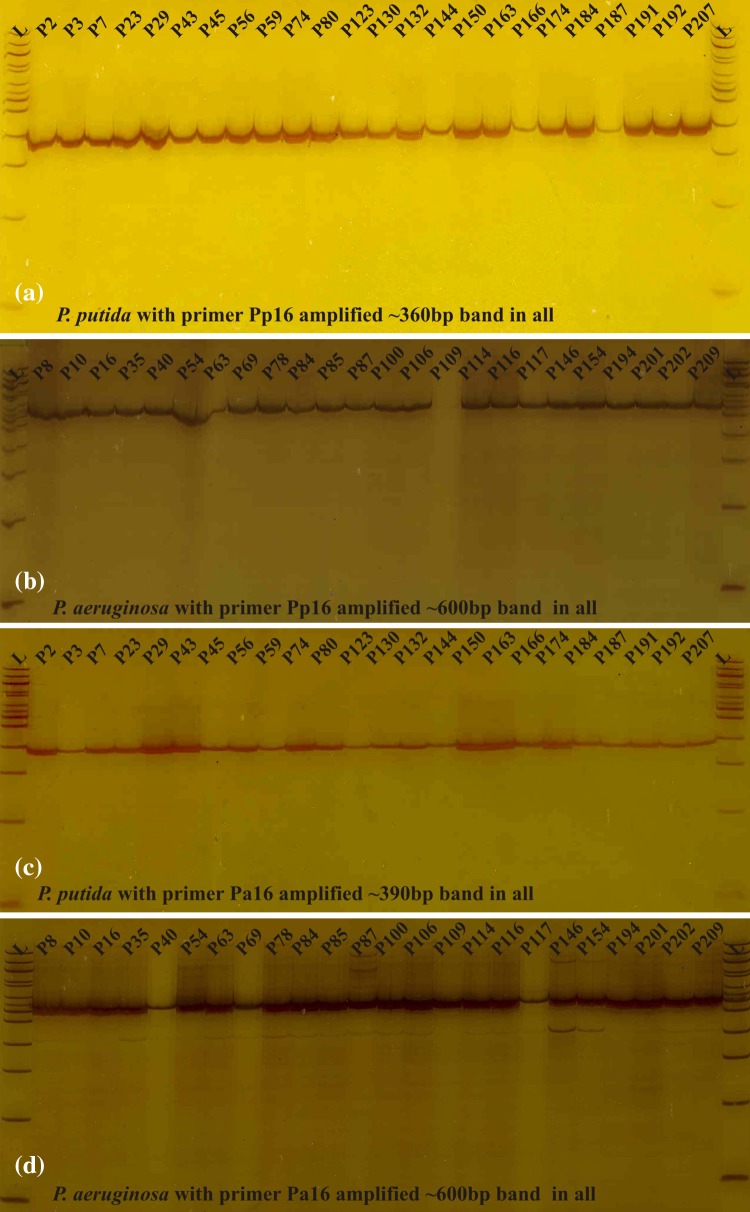
PCR amplification of Pseudomonads using designed 16sRNA-based primers. **a***P. putida* isolates amplified with primers derived from gene HQ317190 (Pp16S) generating ~390 bp bands in all the 24 isolates. **b** Representative *P. aeruginosa* isolates amplified with primers derived from gene HQ317190 (Pp16S) generating ~600 bp bands in all the 24 isolates. **c***P. putida* isolates amplified with primers derived from gene HM067869 (Pa16S) generating ~360 bp bands in all the 24 isolates. **d** Representative *P. aeruginosa* isolates amplified with primers derived from gene HM067869 (Pa16S) generating ~600 bp bands in all the 24 isolates. Representative *P. aeruginosa* isolates were used to show differential amplification of the two species *P. putida* and *P. aeruginosa*

PCR amplification of 16S ribosomal RNA with primers designed from genes HI660468 (Pa49), HM067869 (Pa16S), HQ317190 (Pp16S), EF159157 (Pf16S) and AF869903 (Pf23S) resulted in specific distinguishing amplification products with only two primers from genes HM067869 (Pa16S) and HQ317190 (Pp16S). All 24 isolates resulted in positive reaction with primers designed from genes HM067869 (Pa16S) and HQ317190 (Pp16S). Primer from gene HQ317190 (Pp16S), amplified ~360 bp band in all the *P. putida* isolates and ~600 bp band in all the *P. aeruginosa* isolates. However primer from gene HM067869 (Pa16S) amplified ~390 bp band in all the *P. putida* isolates and ~600 bp band in all the *P. aeruginosa* isolates (Fig. 2a–d). Here *P. aeruginosa* isolates were used as a check to differentiate the two species using aforementioned primers. Absence of amplification with primers designed from genes EF159157 (Pf16S) and AF869903 (Pf23S) of *P. fluorescens* proves that none of the isolates used in the present investigation belonged to the species *P. fluorescens*.

**Fig. 3:**
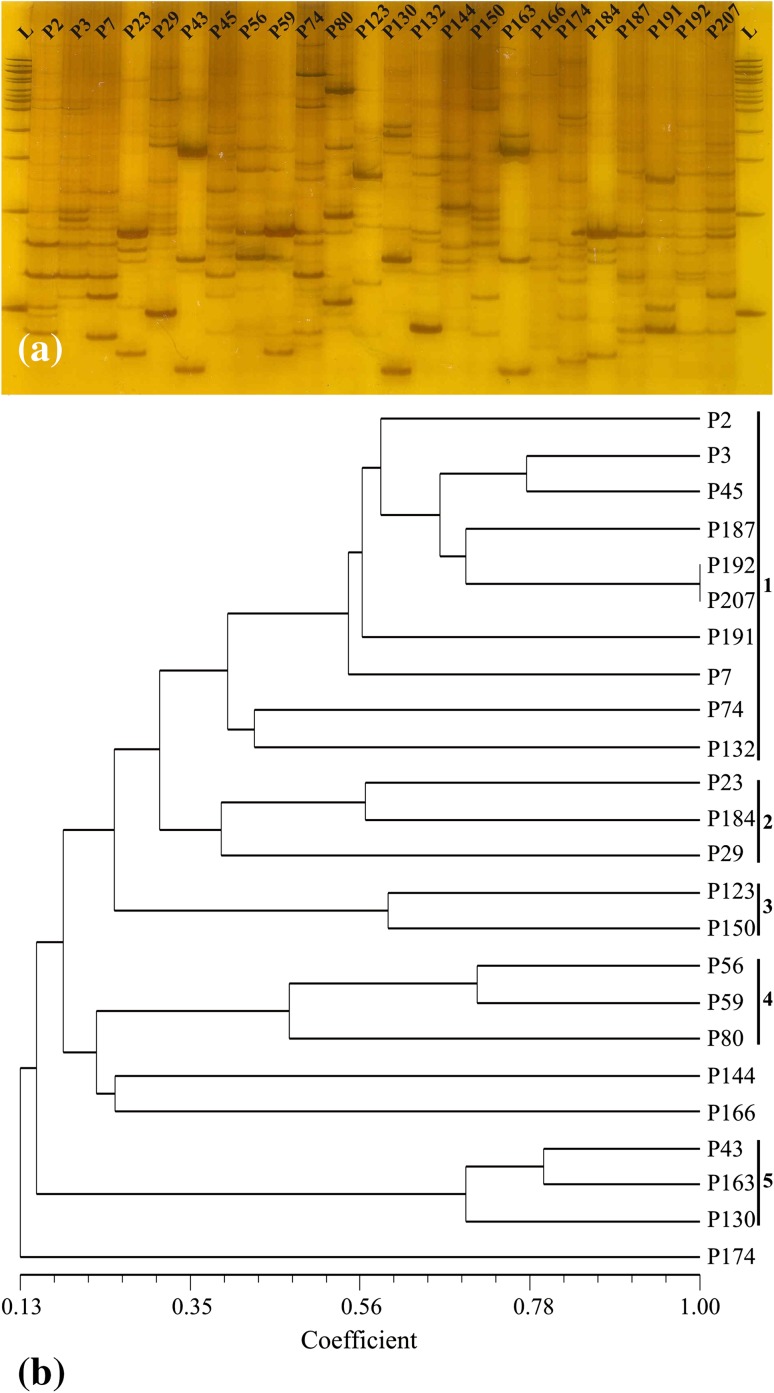
ERIC-PCR-based genotypic analysis of 24 *P. putida* isolates. **a** PCR amplification of 24 *P. putida* isolates generated through ERIC primer. **b** Dendrogram of 24 *P. putida* isolates generated by binary matrix derived from ERIC amplicon**s**

However, a high level of polymorphism was seen in PCR of 24 *P. putida* isolates with ERIC primer. In the present study, the ERIC primer sequence was used in PCR to detect differences in the number and distribution of this bacterial repetitive sequence in the isolates of *P. putida* genomes. The number of bands after PCR amplification of isolates with ERIC primer varied from 5 to 16 with molecular weights between 50 and 1,500 bp (Fig. 3a). The most characteristic products of ERIC amplification for *P. putida* isolates were 310, 200, 180, 165 and 145 bp observed in 13, 16, 16, 13 and 13 out of 24 *P. putida* isolates, respectively. The genetic similarity among 24 *P. putida* isolates ranged from 0.13 to 1.00 (Fig. 3b). The similarity data obtained with ERIC primer identified two major clusters, one of which had only a single *P. putida* isolate P174. Overall the cluster analysis based on the pair-wise coefficient similarity with UPGMA of ERIC-PCR resulted into 5 distinct genomic clusters at similarity coefficient 0.48, viz. groups 1, 2, 3, 4 and 5 consisting of ten (P2, P3, P45, P187, P192, P207, P191, P7, P74, P132), three (P23, P184, P29), two (P123, P150), three (P56, P59, P80) and three (P43, P163, P130) isolates, respectively. Isolates P144 and P166 did not fall into any group. All the isolates exhibited their high degree of genetic variability and distributed into different clusters. This resulted in resolving microdiversity among *P. putida* isolates and significant levels of genomic heterogeneity between strains within and between sites, respectively. Grouping does not appear to be based on geographic origin. The ERIC-PCR fingerprints showed wide variations due to high degree of DNA heterogeneity over all the 24 isolates of *P. putida.* ERIC-PCR confirmed differences in repetitive elements dispersion in *Pseudomonas* genomes and a high degree of genetic variability among phosphate solubilizing *P. putida* isolates. Bacterial isolates with similar biochemical property having approximately common genetic content exhibit molecular diversity. Similar results have been observed by other workers also (McSpadden Gardener 2008; Naik et al. 2008; Kaluzna et al. 2010; Charan et al. 2011).

### In vitro detection of antibiotic-producing *P. putida* isolates using gene-specific primers

**Fig. 4:**
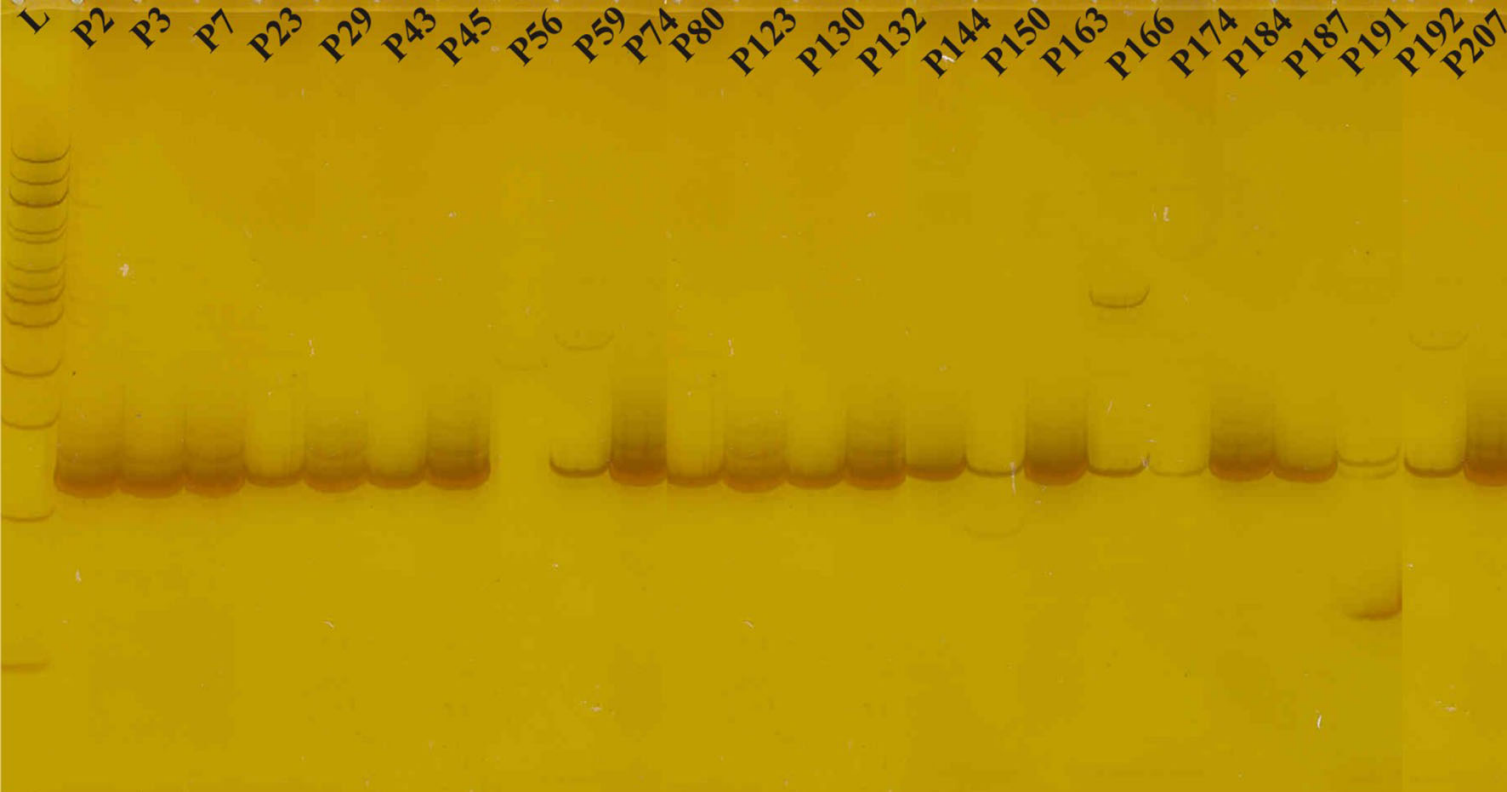
PCR amplification of 24 *P. putida* isolates generated through PhaJ primer showing amplification of ~250 bp in all the isolates except P56

**Fig. 5:**
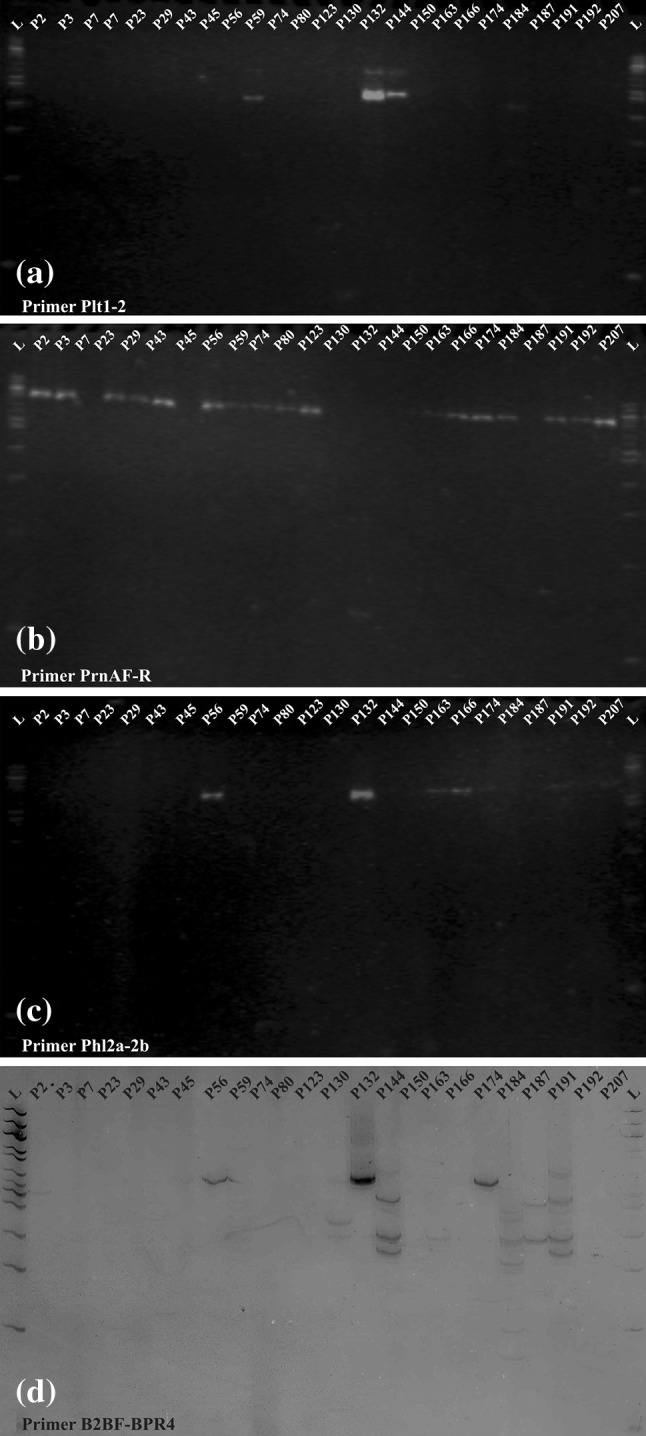
PCR amplification of 24 *P. putida* isolates generated through primer. **a** plt1-2 (Pyrrolnitrin) amplified ~450 bp product in three isolates P56, P132 and P144. **b** PrnAF-R (Pyrrolnitrin) amplified ~1,000 bp fragment in all the isolates except P7, P45, P130, P132, P144, and P150. **c** Phl2a-2b amplified an expected 750 bp fragment in isolates P56 and P132 only; faint bands were observed in P163 and P166 also. **d** B2BF-BPR4 showing amplification of ~629 bp in isolates P56, P132 and P174 and ~350 bp in isolates P130, P144, P163, P174, P184 and P187

The results of the PCR analysis with primer PhaJ (Polyhydroxyalkanoate gene) indicated that a DNA fragment approximately 250 bp in size was obtained in all *P. putida* isolates except P56 (Fig. 4). However, PCR analysis with primers PCA2a-3B (phenazine) and PltBf-r (Pyoluteorin) produced DNA fragments of size 1,400 bp and 800 bp, respectively, in *P. putida* isolate P132 only; all other isolates showed negative results with these primers, i.e., these primers did not yield a PCR product. Pyoluteorin primer PltBf-r amplified another specific 700 bp band in *P. putida* isolates P80 and P132. It may be concluded that these genes were absent in other isolates. Primer plt1-2 (Pyrrolnitrin) amplified ~450 bp product in three isolates P56, P132 and P144 (Fig. 5a). Primer PrnAF-R (Pyrrolnitrin) amplified ~1,000 bp fragment in all the isolates except P7, P45, P130, P132, P144, and P150 (Fig. 5b). 2,4 Diacetylphloroglucinol gene-specific primer Phl2a-2b amplified an expected 750 bp fragment in isolates P56 and P132 only; faint bands were observed in P163 and P166 also (Fig. 5c). 2,4 Diacetylphloroglucinol primer B2BF-BPR4 amplified an expected DNA fragment of approximately 629 bp in three *P. putida* isolates P56, P132 and P174. However DNA fragment of 350 bp was observed in isolates P130, P144, P163, P174, P184 and P187 (Fig. 5d).

Results of PCR analysis with primers of different antibiotic genes showed that *P. putida* isolate P132 isolated from Kanker forest contained genes for phenazine, pyrrolnitrin, pyoluteorin and 2,4 diacetylphloroglucinol along with polyhydroxyalkanoate gene. Isolate P56 amplified 2,4 diacetylphloroglucinol gene with primers Phl2a-2b and B2BF-BPR4 and pyrrolnitrin gene with primers plt1-2 and PrnAF-R but amplification with primer specific for polyhydroxyalkanoate gene (PhaJ) was absent in it. The presence of antibiotic genes can be sued as a suitable marker for screening and selection of bacteria with potential biocontrol activity, in vitro and in situ conditions. However, it may not be necessary that biosynthetic genes for all the antibiotics may be present in all the *Pseudomonas* spp or isolates. Similar results have been reported by Zhang et al. (2006) who used 30 different PCR primers to identify antibiotic-related genes in previously isolated bacteria exhibiting good biocontrol activity. *Pseudomonas* spp. DF41 did not show amplification with primers specific for antibiotic biosynthetic genes encoding PCA, pyrrolnitrin, pyoluteorin and 2,4-DAPG, or for the zwittermicin A self-resistance gene.

It is often difficult and laborious to isolate and identify antibiotic-producing strains from natural environments. However, PCR detection can be a quick alternative to it which depends on the grouping of isolates based on different antibiotic-related genes present. In the present study an attempt was made to characterize *P. putida* isolates for the presence of biosynthetic genes involved in production of different types of antibiotics and related compounds by utilizing multiple primer sets. Significant phenotypic and genotypic differences have been reported during last decades in indigenous populations of fluorescent *Pseudomonas* spp. isolated from the rhizoplane, rhizosphere soil, or nonrhizosphere soil (Lemanceau et al. 1995; Raaijmakers et al. 1997). The genotypic diversity that exists within antibiotic producer *P. putida* isolates can be exploited to improve the rhizosphere competence and biocontrol activity of introduced rhizobacteria. However to manipulate the behavior and activity of biocontrol agents it is necessary to understand the influence of several abiotic and biotic factors on expression of antibiotic biosynthesis.

## Conclusion

The present work provides an insight into the nutritional, biochemical and genetic versatility of 24 *P. putida* isolates. There is variability among isolates for PHB production and release of inorganic phosphate which can be further exploited to select potential isolate for industrial, biocontrol and plant growth promoting applications. All the isolates (except P56) were amplified with polyhydroxyalkanoate gene-specific primer PhaJ but the variability in PHB production may be because of the fact that synthesis of PHB is pathway-dependent and environment-dependent. Also it is a multi-gene-dependent process which necessitates requirement for more number of primers for screening. One of the potential *P. putida* isolate P132 can contribute as a candidate agent for both biocontrol and PGPR applications. Identified as one of the most efficient PHB producer and phosphate solubilizer, in vitro detection of P132 showed the presence of genes for phenazine, pyrrolnitrin, pyoluteorin and 2,4 diacetylphloroglucinol along with polyhydroxyalkanoate. The diversity and adaptation of different isolates in terms of their respective environments provide further insights into their potential applications for the bioremediation of contaminated environments.
